# Effects of Royal Jelly Administration on Endurance Training-Induced Mitochondrial Adaptations in Skeletal Muscle

**DOI:** 10.3390/nu10111735

**Published:** 2018-11-12

**Authors:** Yumiko Takahashi, Kamiyu Hijikata, Kohei Seike, Suguru Nakano, Mai Banjo, Yosuke Sato, Kenya Takahashi, Hideo Hatta

**Affiliations:** Department of Sports Sciences, The University of Tokyo, 3-8-1 Komaba, Meguro-ku, Tokyo 153-8902, Japan; kamiyu.hijikata@gmail.com (K.H.); kseike302043j@gmail.com (K.S.); s.tion@icloud.com (S.N.); mai-banjo@lime.ocn.ne.jp (M.B.); amusnsd@gmail.com (Y.S.); aynekihsahakat@gmail.com (K.T.); hatta@idaten.c.u-tokyo.ac.jp (H.H.)

**Keywords:** endurance training, mitochondrial enzymes, phospho-AMPK, royal jelly, skeletal muscle

## Abstract

We investigated the effect of royal jelly (RJ), a natural secretion from worker bees, on the endurance training-induced mitochondrial adaptations in skeletal muscles of ICR mice. Mice received either RJ (1.0 mg/g body weight) or distilled water for three weeks. The mice in the training group were subjected to endurance training (20 m/min; 60 min; 5 times/week). There was a main effect of endurance training on the maximal activities of the mitochondrial enzymes, citrate synthase (CS), and β-hydroxyacyl coenzyme Adehydrogenase (β-HAD), in the *plantaris* and *tibialis anterior* (*TA*) muscles, while no effect of RJ treatment was observed. In the *soleus* muscle, CS and β-HAD maximal activities were significantly increased by endurance training in the RJ-treated group, while there was no effect of training in the control group. Furthermore, we investigated the effects of acute RJ treatment on the signaling cascade involved in mitochondrial biogenesis. In the *soleus*, phosphorylation of 5′-AMP-activated protein kinase (AMPK) and acetyl-CoA carboxylase (ACC) were additively increased by a single RJ treatment and endurance exercise, while only an exercise effect was found in the *plantaris* and *TA* muscles. These results indicate that the RJ treatment induced mitochondrial adaptation with endurance training by AMPK activation in the soleus muscles of ICR mice.

## 1. Introduction

Mitochondria are important organelles that produce the adenosine triphosphate (ATP) required for the contraction of skeletal muscles. It is well known that endurance exercise promotes mitochondrial biogenesis in skeletal muscles [1]. As the mitochondrial oxidative capacity is related to glycogen-sparing [2], many athletes try to stimulate the mitochondrial adaptations in the skeletal muscles with daily training. Moreover, decreased mitochondrial content has been associated with some metabolic diseases like insulin resistance and obesity [3] and sarcopenia [4]. Therefore, it is of general interest to increase mitochondrial content to maintain good health and a high quality of life. Any additional method to increase the mitochondrial content might be greatly beneficial to both the athletes looking to improve their performance, as well as the common people to maintain their health. In particular, individuals with decreased mobility, obesity, injuries, and other ailments that limit their ability to perform training will benefit from finding an efficient way to increase the mitochondrial content through endurance activities.

Royal jelly (RJ), which is produced by the worker honeybees serves as the food for queen bees for their growth and contains many nutrients including vitamins, minerals, fatty acids, carbohydrates, and proteins/amino acids. Some ingredients contained in RJ have been reported to have a potential for stimulating mitochondrial adaptation in skeletal muscles. For example, supplementation of the protein supplements (including hydrolysate of the protein or peptides) or amino acids enhanced the mitochondrial biogenesis concomitant with the endurance exercise training [5,6]. One possible target of the protein and/or amino acids supplementation is 5′-AMP-activated protein kinase (AMPK), an energy sensor of the cells and a key regulator of mitochondrial biogenesis. Previous reports suggested that leucine [7] and casein peptide [6,8] activate AMPK in skeletal muscles. In addition to amino acids and protein, 10-hydroxy-2-decenoic acid, a unique medium-chain fatty acid present in RJ, was shown to activate AMPK in skeletal muscles [9,10,11]. Therefore, it is possible that RJ treatment is effective for inducing mitochondrial adaptation in skeletal muscles during endurance training.

Mammalian skeletal muscles are mainly composed of three different fiber types that are distinguished through histochemical staining: type I (commonly referred to as slow-twitch and oxidative muscle fiber), type IIA (fast-twitch and oxidative muscle fiber), and type IIB or type IIX (fast-twitch and glycolytic muscle fiber). A previous study showed that the increase in the mitochondrial enzymatic activity by chronic stimulation-induced contraction was greater in the low-oxidative muscles compared to the high-oxidative muscles [12]. This result showed the possibility that the high-oxidative muscles have a higher threshold to induce mitochondrial adaptations compared with the low-oxidative muscles. Meanwhile, casein peptide supplementation with endurance training induced mitochondrial adaptations in the *soleus* muscle (mainly consists type I and IIA fibers), while endurance training alone did not induce mitochondrial adaptation [6]. In that study, casein peptide did not potentiate the mitochondrial adaptations in the *plantaris* muscle (mainly consists type IIB and IIX fibers), in which the maximal activities of mitochondrial enzymes were increased by endurance training alone. Collectively, the possibility exists that RJ treatment combined with endurance training might lead to different results among skeletal muscles with discrete compositions of fibers. Therefore, we investigated the effects of RJ treatment in the soleus muscle (type I: around 35–45%, type IIA: around 35–50%), and the *plantaris* and *tibialis anterior* (*TA)* muscles (the sum of the percentage of type IIB and type IIX fiber types is around 90%) [13,14,15].

In the present study, we investigated the effects of RJ treatment on the mitochondrial adaptations induced by endurance training in skeletal muscles. Moreover, we also examined the acute effects of RJ treatment on the phosphorylation of the signaling cascade proteins related to mitochondrial adaptations in skeletal muscles.

## 2. Materials and Methods

### 2.1. Animals

Nine-week-old male ICR mice were purchased from CLEA Japan, Inc. (Tokyo, Japan) and were housed in a room maintained at 23 °C with three mice per cage. The mice were acclimatized for 1 week. The mice were provided with free access to a standard chow (MF; 3.6 kcal/g, 60% kcal from carbohydrate, 13% kcal from fat, 27% kcal from protein; Oriental Yeast, Tokyo, Japan). All procedures performed in this study involving animals were in accordance with the ethical standards of the Committee on Animal Care and Use, The University of Tokyo. All the protocols of research on animals were approved by this committee (Approval number: 24-4). The dark phase was set to 07:00–19:00, and all the experimental treatments were performed in this phase when the mice were active.

### 2.2. Experimental Procedures

#### 2.2.1. Chronic Experiment

Three days before the first experimental day, all the mice were familiarized with the treadmill exercise at a speed of 20 m/min for 10 min. The mice with similar mean body weights were then divided into four groups: a control sedentary (Con + Sed) group (*n* = 8), a control training (Con + Tr) group (*n* = 6), a RJ-treated sedentary (RJ + Sed) group (*n* = 7), and a RJ-treated training (RJ + Tr) group (*n* = 7). The mice were then housed individually in standard cages. Mice in the RJ-treated group were orally administered with royal jelly (1.0 mg/g body weight) dissolved in distilled water, while the control group mice received distilled water alone every day at 10–12 a.m. The amount of royal jelly used in this study is comparable to that used in a previous study that used RJ mixed with the chow diet [16]. The freeze-dried royal jelly powder standardized to contain a minimum of 3.85% (E)-10-hydroxy-2-decenoic acid and 0.67% of 10-hydroxydecanoic acid was obtained from Yamada Bee Co., Inc. (Okayama, Japan). The nutritional information of freeze-dried royal jelly powder is listed in Table 1. The volume of ingestion was 0.01 mL/g body weight. Mice in the training group started running on the treadmill at a speed of 20 m/min for 60 min after 30–60 min of the oral administration (5 times/week). After three weeks of treatment and 24–28 h post-administration of the final dose, the mice were sacrificed under anesthesia and the tissues were harvested. Blood was drawn from the caudal vena cava and was centrifuged in the presence of heparin. The obtained plasma and the skeletal muscles of the lower hind limb were quickly frozen in liquid nitrogen and stored at −80 °C till further use. 

#### 2.2.2. Acute Experiment

After the acclimatization with treadmill exercise in the same manner as the chronic experiment, the mice with similar mean body weights were divided into four groups: a control sedentary group (*n* = 7), a control exercise group (*n* = 7), RJ-treated sedentary group (*n* = 6), and RJ-treated exercise group (*n* = 7). On the experimental day, the mice were orally administered RJ dissolved in distilled water or distilled water alone as mentioned in the chronic experiment. After 30 min, the mice in the exercise group were subjected to running at 20 m/min for 60 min, while the mice in the sedentary group were kept at rest for 60 min. Following the exercise/rest period, the mice were sacrificed under anesthesia and then tissues were harvested.

### 2.3. Analytical Methods

#### 2.3.1. Muscle Homogenization

Protein isolation from the muscles was performed as described previously [17]. Skeletal muscles were homogenized in a radio immunoprecipitation assay (RIPA) buffer (50 mM Tris-HCl (pH 7.4), 150 mM NaCl, 0.25% deoxycholic acid, 1% NP-40, and 1 mM ethylenediaminetetraacetic acid (EDTA)) supplemented with protease inhibitor mixture (cOmplete Mini, EDTA-free, Roche Applied Science, Mannheim, Germany), and phosphatase inhibitor mixture (PhosSTOP, Roche Applied Science). After centrifugation at 600× *g* for 20 min at 4 °C, the supernatants were collected, and their protein concentrations were determined by the bicinchoninic acid (BCA) assay (Thermo Fisher Scientific, Waltham, MA, USA). The supernatants were diluted with a RIPA buffer.

#### 2.3.2. Mitochondrial Enzyme Activity

The maximal activity of citrate synthase (CS) was determined via the addition of oxaloacetate to a buffer solution containing the muscle homogenates (1:100 dilution), DTNB (5,5′-dithiobis (2-nitrobenzoic acid)), and acetyl coenzyme A (CoA) in 100 mM Tris-HCl buffer (pH = 8.3) [18]. The rate change in absorbance (412 nm) was monitored over 180 s with readings every 30 s. The maximal activity of β-hydroxyacyl CoA dehydrogenase (β-HAD) was determined by addition of acetoacetyl CoA to a buffer solution containing the muscle homogenates (1:100 dilution), nicotinamide adenine dinucleotide (NADH), and EDTA in 50 mM Tris-HCl buffer (pH = 7.0) [19]. The rate change in absorbance (340 nm) was monitored over 180 s with readings every 20 s.

#### 2.3.3. Western Blotting

Western blotting was performed as described previously [17,20]. The protein samples (5–10 μg) and a pre-stained molecular weight marker (Bio-Rad, Hercules, CA, USA) were run on 7.5% sodium dodecyl sulfate–polyacrylamide gel electrophoresis (SDS-PAGE) gels for 60 min at 150 V. The proteins were then transferred from the gels to Hybond-P polyvinylidene difluoride transfer membranes (GE Healthcare Japan, Tokyo, Japan) for 75 min at 100 V. The membranes were then blocked with 5% (*w*/*v*) skim milk or 3% (*w*/*v*) bovine serum albumin (BSA) in tris buffered saline (TBS)-T (20 mM Tris base, 137 mM NaCl, 0.1 mM HCl, and 0.1% (*v*/*v*) Tween 20, pH = 7.5) for 60 min at room temperature. The membranes were incubated with the primary antibody in tris buffered saline with TBS-T (1:1000 or 1:2000 dilution) with 5% BSA overnight at 4 °C. Subsequently, the membranes were incubated for 60 min at room temperature with goat-anti-rabbit IgG (American Qualex, San Clemente CA, USA) in TBS-T (1:4000 dilution). The proteins were detected using Pierce ECL Western Blotting Substrate (Thermo Fisher Scientific) and visualized using the ChemiDoc system (Bio-Rad). Densitometric analyses of the captured images were performed using Bio-Rad Quantity One software (version 4.6.1). All the membranes were stained with Ponceau-S solution (P7170-1L; Sigma-Aldrich, St. Louis, MO, USA) to ensure equal loading of the proteins. The antibodies used in this study were anti-AMP-activated protein kinase (AMPK, #5832; Cell Signaling Technology [CST] Japan, Tokyo, Japan), anti-phosphorylated AMPK (Thr172, #2535; CST), anti-acetyl-CoA carboxylase (ACC, #3676; CST), anti-phosphorylated ACC (Ser79, #11818; CST), anti-p38 mitogen-activated protein kinase (p38 MAPK, #9212; CST), and anti-phosphorylated p38 MAPK (Thr180/Tyr182, #9211; CST).

### 2.4. Statistical Analysis

All values were expressed as the mean ± standard error. Prism 6 software (GraphPad Software, San Diego, CA, USA) was used for the statistical analyses. Two-way analysis of variance (RJ treatment × endurance training or RJ treatment × endurance exercise) was performed to determine the differences in each parameter. If an interaction was observed, the Tukey–Kramer multiple-comparison test was performed. Statistical significance was set at *p* < 0.05.

## 3. Results

### 3.1. Chronic Experiment

#### 3.1.1. Food Consumption and Body Weight

The mice were weighed post-treatment and the results suggested that neither the endurance training nor the RJ treatment had a significant effect on their final body weight at the end of the experiment. We also observed that there was no significant effect on the food consumption of the mice upon endurance training and the RJ treatment (Table 2).

#### 3.1.2. Maximal Activities of Mitochondrial Enzymes

**Figure 1:**
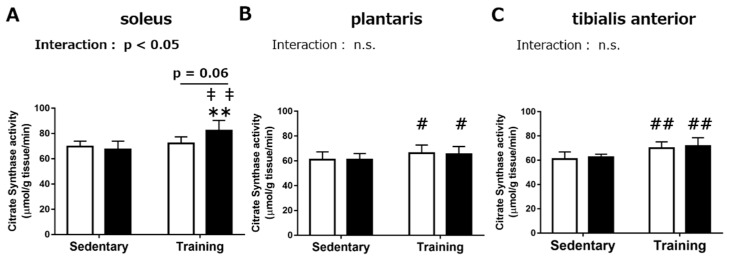
Maximal activity of citrate synthase in the *soleus* (**A**), *plantaris* (**B**), and *tibialis anterior* muscle (**C**) of the mice after treatment with royal jelly (RJ-treated group; black) or distilled water (Control group; white), with or without endurance training for three weeks (Chronic experiment). Values represent mean ± standard error. *n* = 6–8 per group. #*p* < 0.05 and ##*p* < 0.01, main effect of endurance training. **p < 0.01, statistical significance vs the RJ-treated sedentary group. ‡‡*p* < 0.01, statistical significance between the control sedentary group. n.s.: not significant.

In the soleus muscle, the results from the biochemical assay to measure the maximal CS activity demonstrated a significant interaction between the RJ treatment and endurance training (Figure 1A, *p* < 0.05). Therefore, we performed the Tukey–Kramer multiple-comparison test. In the RJ group, the maximal CS activity was significantly increased upon endurance training (*p* < 0.01), while no significant effect of endurance training was observed in the control group. The maximal CS activity in the RJ-treated training group was significantly higher than that in the control sedentary group (*p* < 0.01) and tended to be higher than that in the control training group (*p* = 0.06). There was a significant positive effect of endurance training on the maximal CS activity in the *plantaris* (*p* < 0.05) and *tibialis anterior* (*TA*, *p* < 0.01) muscles, while no significant effect upon RJ treatment was found (Figure 1B,C).

**Figure 2:**
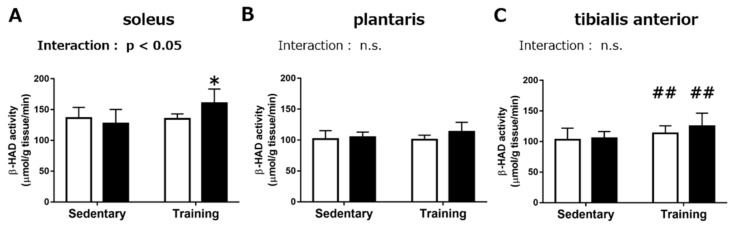
Maximal activity of β-hydroxyacyl CoA dehydrogenase in the *soleus* (**A**), *plantaris* (**B**), and *tibialis anterior* muscle (**C**) of mice that treated with royal jelly (RJ-treated group; black) or distilled water (Control group; white), with or without endurance training for three weeks (Chronic experiment). Values represent mean ± standard error. *n* = 6–8 per group. ##*p* < 0.01, main effect of endurance training. *p < 0.05, statistical significance vs the RJ-treated sedentary group. n.s.: not significant.

Next, we measured the maximal activity of β-hydroxyacyl CoA dehydrogenase (β-HAD), which catalyzes the rate-limiting step of β-oxidation of long-chain fatty acids. In the *soleus*, we observed a significant interaction between RJ treatment and endurance training (*p* < 0.05). The Tukey–Kramer multiple-comparison test indicated that the maximal activity of β-HAD in the RJ-treated training group was significantly higher than that in the RJ-treated sedentary group (*p* < 0.05), while no significant difference between the control training group and the control sedentary group was observed (Figure 2A). There was a significant positive effect of endurance training in the TA muscle (*p* < 0.01). No significant effect of RJ treatment was observed in the *plantaris* and *TA* muscle (Figure 2B,C).

### 3.2. Acute Experiment

#### Phosphorylation Status of the Proteins Involved in Mitochondrial Biogenesis

**Figure 3:**
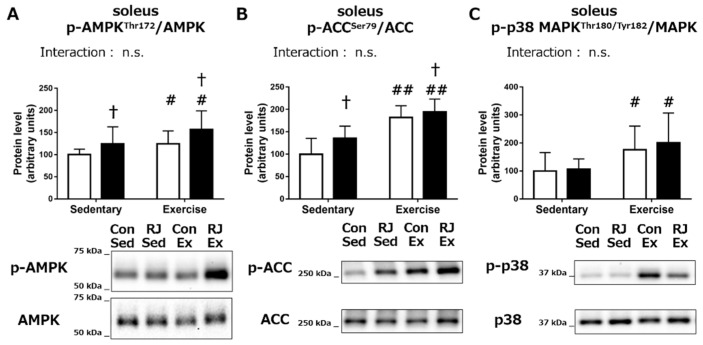
(**A**) 5′-AMP-activated protein kinase (AMPK), (**B**) acetyl-CoA carboxylase (ACC), and (**C**) p38 mitogen-activated protein kinase (p38 MAPK) phosphorylation status in the *soleus* muscle of mice that treated with a single dose of royal jelly (RJ-treated group; black) or distilled water (Control group; white) and with or without an endurance exercise (Acute experiment). Values represent mean ± standard error. *n* = 6–7 per group. #*p* < 0.05 and ##*p* < 0.01, main effect of endurance exercise. †*p* < 0.05, main effect of RJ treatment. n.s.: not significant. Con: control; Sed: sedentary; RJ: royal jelly; Ex: endurance exercise.

In order to study the role of RJ treatment in mitochondrial biogenesis, we further investigated the effects of acute RJ treatment combined with endurance exercise on the phosphorylation status of the crucial signaling proteins. In the *soleus*, phosphorylation of AMPK was additively increased by a single RJ treatment (*p* < 0.05) and a 60 min of endurance exercise (*p* < 0.05, Figure 3A). Similarly, the levels of phospho-ACC, the substrate for AMPK, was also found to be increased following RJ treatment (*p* < 0.05) and endurance exercise (*p* < 0.01, Figure 3B). The phosphorylation of p38 MAPK, another crucial signaling molecule involved in mitochondrial biogenesis, was also significantly increased by endurance exercise (*p* < 0.05). However, no significant change in the p-p38 MAPK levels was noted upon RJ treatment (Figure 3C).

**Figure 4:**
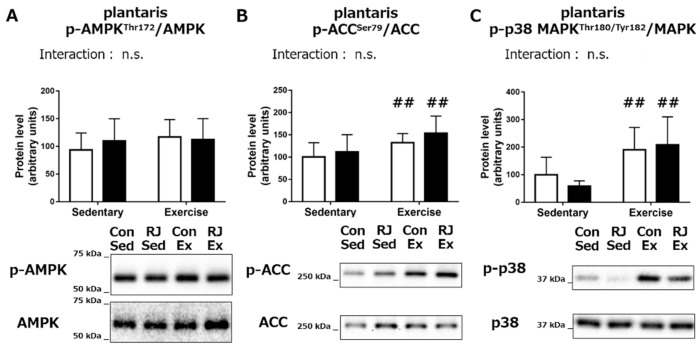
(**A**) AMPK, (**B**) ACC, and (**C**) p38 MAPK phosphorylation status in the *plantaris* muscle of mice that was treated with a single dose of royal jelly (RJ-treated group; black) or distilled water (Control group; white) and with or without an endurance exercise (Acute experiment). Values represent mean ± standard error. *n* = 6–7 per group. ##*p* < 0.01, main effect of endurance exercise. n.s.: not significant.

In the *plantaris* muscle, we did not observe any significant change in the phosphorylation status of AMPK, ACC, or p38 MAPK upon RJ treatment. Conversely, the effect of endurance exercise on the phosphorylated status of ACC (*p* < 0.01) and p38 MAPK (*p* < 0.01) was observed (Figure 4A–C).

**Figure 5:**
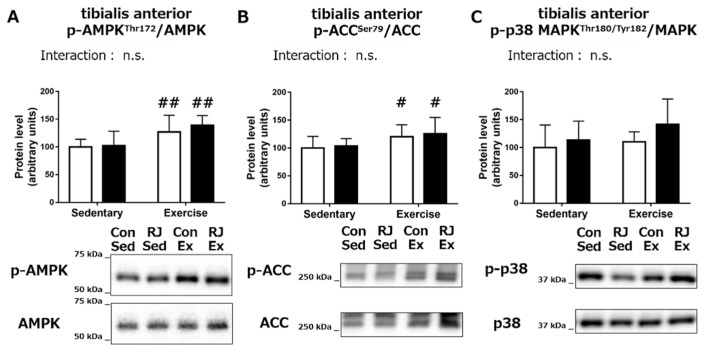
(**A**) AMPK, (**B**) ACC, and (**C**) p38 MAPK phosphorylation status phosphorylation status in *tibialis anterior* muscle of mice that treated with a single dose of royal jelly (RJ-treated group; black) or distilled water (Control group; white) and with or without an endurance exercise (Acute experiment). Values represent mean ± standard error. *n* = 6–7 per group. #*p* < 0.05 and ##*p* < 0.01, main effect of endurance exercise. n.s.: not significant.

In the *TA* muscle, the main effect of endurance exercise on p-AMPK (*p* < 0.01) and p-ACC (*p* < 0.05) were noted but there was no change in their levels upon RJ treatment (Figure 5A,B). No significant main effect of endurance exercise or RJ treatment on the phosphorylation of p38 MAPK was observed in the *TA* muscle (Figure 5C). These results indicate that a differential phosphorylation status of the proteins involved in the mitochondrial biogenesis in different skeletal muscles upon RJ treatment and endurance training.

## 4. Discussion

The main finding of this study is that the oral royal jelly administration had a significant positive effect on inducing the increase in maximal activity of mitochondrial enzyme by endurance training in the *soleus* muscle, which mainly consists of type I and IIA fibers, while no significant effect of RJ treatment on mitochondrial adaptation was observed in the *plantaris* and *TA* muscles, which predominantly consist of type IIB/IIX fibers. Acute RJ treatment and endurance exercise additively increased the phosphorylation of AMPK and ACC, a downstream substrate of AMPK, in the *soleus* muscle, while no effect of acute RJ treatment was noted in the *plantaris* and *TA* muscles.

As the maximal activity of CS, which catalyzes the rate-limiting step of the tri-carboxylic acid (TCA) cycle, is strongly associated with the mitochondrial content in skeletal muscles [21], it is generally used as an indicator of mitochondrial oxidative capacity. In the *plantaris* and *TA* muscles, the endurance training had a significant main effect on the maximal CS activity. However, no effect of RJ treatment was observed in these muscles. On the other hand, we found that the RJ treatment concomitant with the endurance training increased the maximal CS activity in the *soleus* muscle, while no change was observed in the control training group. We observed a similar trend in the maximal activity of β-HAD, which catalyzes the rate-limiting step of fatty acid β-oxidation. These results suggest that the endurance exercise was enough to induce mitochondrial enzymatic adaptation in fast-twitch fiber dominant muscles, while it was contrary in the high-oxidative muscles. A previous study showed that the increase in the mitochondrial enzymatic activity by chronic stimulation-induced contraction was greater in the low-oxidative muscles compared to the high-oxidative muscles [12]. Therefore, the possibility exists that the high-oxidative muscles have a higher threshold to induce mitochondrial adaptation than the low-oxidative muscles. In the present study, although the endurance exercise induced significant positive effects on the phosphorylated states of the signaling proteins involved in mitochondrial biogenesis in the *soleus*, additional stimulation by RJ treatment was needed to induce the increase in the maximal mitochondrial enzymatic activities.

Some components contained in RJ such as amino acids/proteins [6,8], particularly leucine [7], and 10-hydroxy-2-decenoic acid [9,10,11] which is the unique fatty acid present in RJ have been reported to induce the activation of AMPK, a major mediator of the mitochondrial biogenesis, in skeletal muscles, or the myotubes. Meanwhile, RJ contains several components that have been suggested to have antioxidant property: 10-hydroxydecanoic acid; free amino acids, such as proline, cystine, and cysteine; flavonoids; and phenolic compounds [22,23,24]. Previous studies suggested that oxidative stress plays an important role in AMPK activation in skeletal muscle and then the use of antioxidants might affect AMPK activation [25,26]. Moreover, mitochondrial oxidative stress has been suggested to be involved in calcium handling [27] and influence an activation of Ca^2+^/calmodulin-dependent kinase kinase (CaMKK), which is one kind of upstream kinases of AMPK [28]. Therefore, we examined the effects of a single dose of RJ and endurance exercise on the activation of the AMPK signaling cascade. In the soleus, RJ treatment additively increased the phosphorylation levels of AMPK and ACC concomitant with endurance exercise. We also measured the phosphorylation status of p38 MAPK, an activator of mitochondrial biogenesis as well as AMPK, and there was no effect of RJ treatment in the *soleus*. Collectively, the effect of RJ treatment on the endurance training-induced increase in the maximal activities of mitochondrial enzymes in *soleus* seems to be mediated by the AMPK signaling amplification. On the other hand, no effect of RJ treatment was observed on the phosphorylation levels of AMPK signaling cascade proteins in *plantaris* and *TA* muscles. Therefore, the effect of RJ treatment on the activation of AMPK signaling was specific to the soleus muscles, which predominantly consist of type I and type IIA muscle fibers. Regarding amino acids/proteins, a previous study that was focused on investigating the effect of oral casein peptide administration indicated results similar to the present study. They reported that casein peptide treatment increased the phosphorylation in the AMPK regulatory site in the soleus, while no effect was observed in the *plantaris* [6,8]. However, the mechanism behind the effect of these nutrients on the differential activation levels of AMPK activation in different skeletal muscles with distinct fiber type composition has not yet been elucidated. Leucine is one of the potential activators of AMPK in skeletal muscles [7]. A previous study showed that the uptake of glutamate, which indicates a simultaneous efflux against leucine, was lower in the soleus compared with those in *flexor digitorum brevis* and *epitrochlearis* muscles, which are predominantly made of fast-twitch fibers [29]. As the intracellular glutamine contributes to the leucine uptake as an exchange material, thus the lower glutamine uptake in the *soleus* would not induce leucine uptake into muscles. Moreover, a recent study demonstrated that the expression level of the L-type amino acid transporter 1 (LAT1), which is thought to be a primary transporter of neutral amino acids including branched chain amino acids (leucine, valine, isoleucine), was higher in the type II fiber than in the type I fiber [30]. Therefore, it is quite unlikely that the observed differential response to RJ treatment in *soleus*, *plantaris*, and *TA* muscles is due to the difference in amino acids transport into the cells (particularly leucine). To date, no study has compared the effect of 10-hydroxy-2-decenoic acid and other components of RJ on AMPK activation in different muscle fibers. Further studies are warranted to elucidate the mechanism behind the differential response to the RJ components in different skeletal muscles with discrete types of muscle fibers.

In the present study, a positive effect of RJ treatment on endurance training-induced mitochondrial adaptations was observed in the mice *soleus* muscle, which is mainly composed of type I (around 35–45%) and type IIA (around 35–50%) fibers [13,14,15]. In human *vastus lateralis* muscles, which is frequently used in human studies, the percentage of type I and type IIA fibers correspond to those in the mice soleus muscle [31,32]. Therefore, the possibility exists that effect of RJ treatment combined with endurance training might be observed in human skeletal muscles. We provided mice with 1.0 mg/g body weight of RJ for three weeks. It is recommended that the dose of drug or nutrient is converted across animal species based on the body surface area [33]. According to the calculation method proposed by Reagan-Shaw et al. [33], an estimated human equivalent dose of RJ used in the present study is 81.1 mg/kg body weight (4.9 g for 60 kg person). This amount of RJ intake corresponds to that used in the previous study, in which elderly people received RJ for one year [34]. In that study, no side effect of RJ intake was reported. To our knowledge, no study has examined the effect of RJ treatment on endurance training-induced mitochondrial adaptations in human. Further research is needed to elucidate whether three weeks of RJ treatment with endurance training shows beneficial effect for inducing mitochondrial adaptations in human.

## 5. Conclusions

The present study demonstrates that a RJ treatment stimulates an endurance exercise-induced increase in the maximal activities of citrate synthase and β-hydroxyacyl CoA dehydrogenase in the *soleus* muscles (mainly consists type I and type IIA fibers). The study also reveals the possible role of AMPK activation upon RJ treatment on mitochondrial adaptations concomitant with endurance exercise. Moreover, we also demonstrated the difference in response to RJ treatment according to the difference in the composition of muscle fibers.

## Figures and Tables

**Table 1:** Composition of the freeze-dried royal jelly powder (per 100 g)

**Macronutrients**		**Royal Jelly Specific Fatty Acids**	
Water	2.2 g	(E)-10-hydroxy-2-decenoic acid	4.3%
Protein	40.9 g	10-hydroxydecanoic acid	0.94%
Fat	5.5 g		
Minerals	2.8 g		
Ash	2.8 g		
Carbohydrate	48.6 g		
Calories	408 kcal		
**Amino Acids**		**Free Amino Acids**	
Arginine	2.01 g	Free Arginine	0.07 g
Lysine	2.83 g	Free Lysine	0.67 g
Histidine	1.07 g	Free Histidine	0.02 g
Phenylalanine	1.70 g	Free Phenylalanine	0.004 g
Tyrosine	1.65 g	Free Tyrosine	0.006 g
Leucine	2.88 g	Free Leucine	0.005 g
Isoleucine	1.83 g	Free Isoleucine	0.005 g
Methionine	1.03 g	Free Methionine	not detected
Valine	2.09 g	Free Valine	0.01 g
Alanine	1.20 g	Free Alanine	0.01 g
Glycine	1.29 g	Free Glycine	0.01 g
Proline	1. 69 g	Free Proline	0.67 g
Glutamic acid	3.84 g	Free Glutamic acid	0.14 g
Serine	2.31 g	Free Serine	0.004 g
Threonine	1.72 g	Free Threonine	0.002 g
Aspartic acid	6.82 g	Free Aspartic acid	0.006 g
Tryptophan	0.49 g	Free Tryptophan	not detected
Cysteine	0.40 g	Free Cysteine	not detected

**Table 2:** Body weight and food consumption of mice

Title	Con + Sed Group	RJ + Sed Group	Con + Tr Group	RJ + Tr Group
Initial body weight (g)	38.6 ± 0.7	38.1 ± 0.9	37.7 ± 0.8	38.4 ± 0.5
Final body weight (g)	40.5 ± 0.8	40.2 ± 0.7	39.1 ± 0.9	39.3 ± 0.6
Δ body weight (g)	2.0 ± 0.3	2.0 ± 0.5	1.4 ± 0.6	0.9 ± 0.7
Total food consumption (kcal/g body weight)	9.1 ± 0.2	9.5 ± 0.4	9.1 ± 0.5	9.1 ± 0.5

Values represent mean ± standard error. *n* = 6–8 per group. Con: control; Sed: sedentary; RJ: royal jelly; Tr: training; Δ body weight: change in body weight during Chronic experiment.
